# A Deep Non-negative Matrix Factorization Model for Big Data Representation Learning

**DOI:** 10.3389/fnbot.2021.701194

**Published:** 2021-07-20

**Authors:** Zhikui Chen, Shan Jin, Runze Liu, Jianing Zhang

**Affiliations:** School of Software, Dalian University of Technology, Dalian, China

**Keywords:** non-negative matrix factorization, deep representation learning, denoising autoencoder, interpretability, supervisor network

## Abstract

Nowadays, deep representations have been attracting much attention owing to the great performance in various tasks. However, the interpretability of deep representations poses a vast challenge on real-world applications. To alleviate the challenge, a deep matrix factorization method with non-negative constraints is proposed to learn deep part-based representations of interpretability for big data in this paper. Specifically, a deep architecture with a supervisor network suppressing noise in data and a student network learning deep representations of interpretability is designed, which is an end-to-end framework for pattern mining. Furthermore, to train the deep matrix factorization architecture, an interpretability loss is defined, including a symmetric loss, an apposition loss, and a non-negative constraint loss, which can ensure the knowledge transfer from the supervisor network to the student network, enhancing the robustness of deep representations. Finally, extensive experimental results on two benchmark datasets demonstrate the superiority of the deep matrix factorization method.

## 1. Introduction

Nowadays, deep representations have been attracting increasing attention in various domains, such as image segmentation, pattern mining, and fault detection, in which hidden patterns of data can be well-captured with the help of edge-cutting deep architectures (Sengupta et al., 2020; Luo P. et al., 2021). However, the interpretability of deep representations is still a vast challenge that greatly limits real-world applications. For instance, if parts of the deep representation of a face image can be explained as eyes and ears, it will be easy to manipulate face images by replacing corresponding parts. For another instance, people may agree with the applications of deep representations, irrelevant to themselves, but they shall require to know how deep neural networks make decisions on relevant areas, such as medical treatment and autonomous driving. To solve the challenge of interpretability, many researchers tried to excavate meanings of deep representations, but could only achieved some shallow explanations that deep representations contain spatial and temporal relation information of data.

Non-negative matrix factorization (NMF), as an explicable model, factorizes the original data matrix by the product of a base matrix and a weight matrix (Lee and Seung, 1999; Févotte and Idier, 2011). To be specific, given the original data matrix **X** composed of data vectors **x**_*i*_ in data space, **X** is equal to the product of the identity matrix **I** and **X**. More generally, **X** is equal to the product **PP**^−1^**X** consisting of an invertible matrix **P**, the inverse matrix **P**^−1^ and **X**, since the invertible matrix **P** is a set of base vectors of the original data space. Suppose data vectors are approximatively distributed in a subspace of the original data space, the base vectors which are not in that subspace are useless for representation, and they can be ignored to save the storage space and computation resources, which benefits many fields such as fog computing (Laghari et al., 2021), image transmission with little loss of QoE/QoS (Laghari et al., 2018; Karim et al., 2021), and detecting and controlling street crimes by drones (Karim et al., 2017). It is the basic idea behind matrix factorization methods to only use fewer base vectors and weight factors for approximatively representing the same information. As a representative matrix factorization algorithm, NMF requires that the original data matrix, base matrix, and weight matrix follow the non-negative constraint, that is, all elements of the three matrices should not be the negative value. Thus, the elements of those matrices can be used to denote physical quantities in the nature whose values are non-negative, such as length, weight, and speed. In other words, the non-negative constraint ensures the interpretability of NMF such that data in the original data matrix can be explained as the weighted sum of low-dimensional base vectors in the base matrix, and the weights are represented by the weight matrix.

To explore the interpretability of deep representations, a lot of researches combine NMF with deep neural networks. A typical method is to directly combine NMF and the autoencoder of the encoder-decoder architecture. Specifically, the encoder transforms input data into a low-dimensional feature space, while the decoder maps data features back to the original data space by reconstructing the input data. The autoencoder is good at learning deep representations of data. After obtaining deep representations, NMF is conducted on the deep representations. This method can achieve good performance, since it uses the autoencoder to extract deep representations of data and obtains the interpretability by the weighted sum of physical quantities with the non-negative constraint, resulting in the final explicable deep representations.

However, the existing methods depend on the two-stage manner to combine NMF and the autoencoder. In those methods, autoencoder is only used as the feature extractor to model deep low-dimensional representations of data, and NMF only works on the extracted deep representations. Those two parts work separately and do not mutually guide the learning of each other, which limits the performance of the existing methods. Furthermore, those above methods do not fully consider the noise hidden in the input data which obstructs the robustness of deep representations in real world applications.

To solve those challenges, in this paper, we propose a deep matrix factorization model with non-negative constraints (DDNMF) for learning deep robust interpretable representations of data. In details, a deep knowledge distillation architecture is devised with a supervisor network and a student network, which can address the weakness of noise and the two-stage combination of NMF and the autoencoder. Besides, an interpretability loss is introduced to train parameters of the deep matrix factorization model, which is composed of a symmetric loss that guides the supervisor network, an apposition loss that teaches the student network, and a non-negative constraint loss that learns the interpretable knowledge. The representations learned by proposed model can be used for various downstream tasks such as clustering and classification. Experimental results on standard datasets can demonstrate the effectiveness of the proposed model.

The rest of this paper is organized as follows: section 2 introduces preliminaries and related works about non-negative matrix factorization and deep neural networks. Section 3 proposes the deep denoising non-negative matrix factorization. The experimental evaluation on standard datasets is shown in section 4. And section 5 concludes the proposed method and declares the direction of future work.

## 2. Preliminaries and Related Works

NMF is a well-known matrix factorization method (Jia et al., 2021; Luo X. et al., 2021). It represents the original data matrix **X** using the product of a base matrix **H** and a weight matrix **W**: **X** ≈ **WH**, where **X** ∈ *R*^*m*×*n*^, **W** ∈ *R*^*m*×*r*^, and **H** ∈ *R*^*r*×*n*^. The row and column of original data matrix **X** stand for the dimension and number of data samples, respectively. Through NMF, the dimension of data *m* is changed as *r*, the dimension of the feature. There usually exists *r* < *m* and *r* × (*m* + *n*) < *m* × *n*, so the result of NMF reduces the feature dimension and saves the storage space and computation resource. At the same time, all elements of these three matrices should be no less than zero: **X** ≥ 0, **W** ≥ 0, **H** ≥ 0. Through choosing special learning rates of the gradient descent method, Lee and Seung (1999) derived the multiplicative update formulas of NMF which optimize the base matrix **H** and the weight matrix **W** with keeping their non-negative properties by the multiplication and division operations between non-negative elements.

However, NMF is a simple linear factorization method such that it cannot capture the non-linear relation hidden in data. In the past decade years, much attention was paid to enhance NMF for fitting non-linear complex data or representations. For example, Zhang et al. (2006) proposed a kernel-based NMF algorithm in which NMF was conducted on compact representations transferred by kernels. Buciu et al. (2008) introduced a polynomial NMF with the help of the non-linear polynomial kernel mapping contributing to the correlation of the high-order of basis image features. The kernel-based NMF can improve representations produced by NMF with the kernel mapping (Duong et al., 2014). Those methods fit the non-linear relation between data by kernels.

Nowadays, deep learning has achieved great progress on extracting deep representations for various downstream tasks. For example, Vaswani et al. (2017) deployed the attention mechanism in the proposed model named Transformer without the operations of convolutions and recurrences, and led a trend of applying Transformer in different fields of machine learning, such as, computer vision, natural language processing. Guo et al. (2020) proposed DeepANF for the prediction of chromatin accessibility by attention mechanism, gated recurrent units and convolutions, learning the deep representations of DNA sequences.

To further enhance NMF, some deep learning models are embedded in NMF. For instance, Ye et al. (2018) proposed a deep autoencoder-like non-negative matrix factorization by combining an encoder of matrix factorization and a decoder of the symmetric architecture. Bhattamishra (2018) designed a deep probabilistic NMF on the basis of autoencoders, in which a probabilistic NMF is built on deep representations of a deep autoencoder with an alternate manner of learning. Bando et al. (2018) proposed a deep variational NMF by interpreting the spectrogram of input data as the sum of a speech spectrogram and a non-negative noisy spectrogram, which is modeled as the variational prior distribution. Ren et al. (2019) introduced an end-to-end deep NMF architecture with non-negative constraints and a factorization loss conducted on middle layers. Those deep NMFs can further learn non-linear correlations hidden in data. However, in those deep NMFs, deep learning and NMF work in the separate manner. Furthermore, those deep NMFs cannot well take noise into consideration.

## 3. Methodology

### 3.1. The Deep Matrix Factorization Model

**Figure 1:**
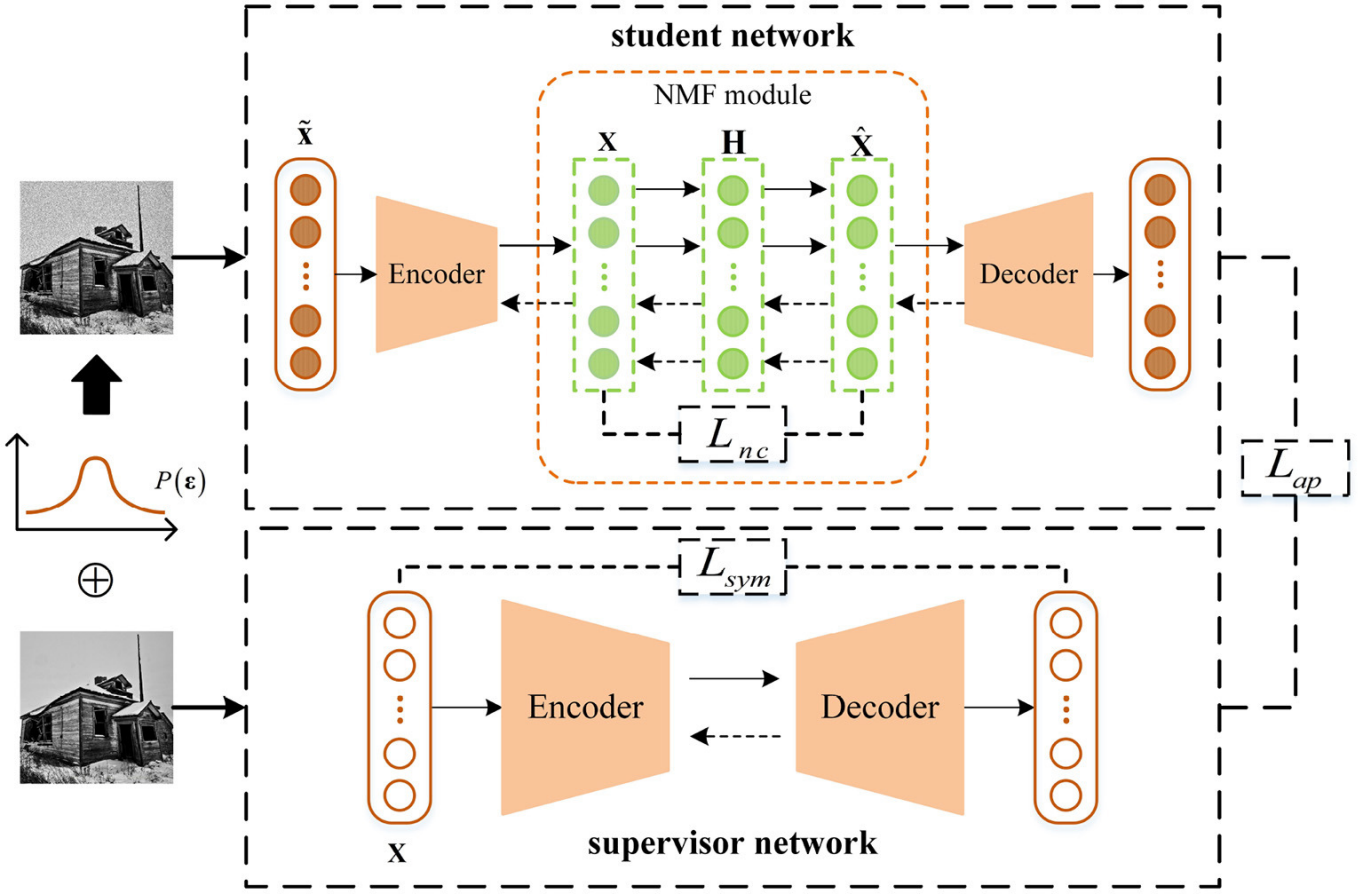
The architecture of DDNMF.

As shown in Figure 1, DDNMF contains two parts: a supervisor network and a student network. Each of the two networks includes an encoder and a decoder. The difference between the supervisor network and the student network is that a NMF module is inserted between the encoder and the decoder of the student network.

As mentioned above, deep neural network is good at extracting deep representations in an unsupervised manner by reconstructing inputs. The supervisor network in DDNMF works for the same goal as standard deep neural networks. Thus, the output of each layer is computed as follows:

(1) $$h_{i}^{\left(l\right)}=f^{\left(l\right)}(W^{\left(l\right)}h_{i}^{\left(l-1\right)}+b^{\left(l\right)}),l=1,\ldots ,L$$

where $h_{i}^{\left(l\right)}$ is the output feature of the *l*-th layer corresponding to *i*-th input data **x**_*i*_. Specially, when the layer is the input layer, $x_{i}=h_{i}^{\left(0\right)}$. *f*^(*l*)^, **W**^(*l*)^, and **b**^(*l*)^ are the activation function, weight matrix, and bias vector of the *l*-th layer, respectively. *L* is the total number of layers in the supervisor network. It must be an even number due to the symmetry architecture of an encoder and a decoder with the same structure. The size of the input feature in the *l*-th layer equals to the size of the output feature in the *L*−*l*+1-th layer. And the sizes of the weight matrix and the bias vector in a layer are decided by the input and output sizes of the layer. Thus, there is a transposition relationship between the weight matrices of symmetric layers.

Like the supervisor network, the encoder and decoder in student network extract the deep representations of input data. The input data are added with noise to mimic the real world data as follows:

(2) $${\tilde{x}}_{i}=x_{i}+\varepsilon_{i},\varepsilon_{i}\sim P\left(\varepsilon \right)$$

where ε_*i*_ is the noise vector sampled from the probability distribution *P*(ε). This imitation gives the student network a chance of learning how to remove noise mixed in data. Thus, the supervisor network receives the input data and outputs the same data, while the student network receives the noisy data and outputs the noise-free data.

In normal encoder-decoder structure, the output of encoder means the input of decoder. But between the encoder and decoder of the student network, there is a NMF module for bringing the interpretability to DDNMF. The NMF module receives and factorizes the encoded representation, then recovers the representation by matrix multiplication for decoding in next step. Like vanilla NMF, it follows the non-negative constraint and matrix factorization pattern. However, different from previous work which combined NMF with deep neural network by two stages, DDNMF does not take them apart for two-stage training, but always fix the NMF module between the encoder and decoder as an inseparable part of the student network. And for fitting non-linear relation hidden in the latent feature, non-linear sigmoid activation function is introduced in the NMF module. The non-negative constraint and non-linear fitting ability are implemented as:

(3) $$\begin{array}{l} H_{i}=\sigma (W^{\prime }X_{i})\\ {\hat{X}}_{i}=ReLU(WH_{i}) \end{array}$$

where **X**_*i*_ represents the input matrix consisting of the output of encoder. ${\hat{\text{X}}}_{i}$ represents the recovered matrix composed of the input of decoder. **W**′ has the same size as the transpose matrix of **W** to transform **X**_*i*_ into the space of **H**_*i*_. The non-negative constraint is imposed on the recovered matrix ${\hat{\text{X}}}_{i}$ by Rectified Linear Unit (ReLU) activation function *ReLU*(·) [*ReLU*(*x*) = *max*(*x*, 0)] and base matrix **H**_*i*_ is ruled with non-linear sigmoid transformation function σ(·) which maps all input to the value between zero and one, which is also non-negative.

As shown in Figure 1, the original input data are input to two networks. In the supervisor network, data are transformed into hidden feature space, and features are recovered to data space. In the student network, data are added by random noise first, then processed by feature extracting and NMF, and finally reconstructed as the outputs. In this architecture, the NMF module brings the interpretability, and two networks extract deep hidden information. Among them, the student network focuses on learning feature without disturbance, and the supervisor network helps it keep the insight for the latent information and structure. The next subsection will explain how the proposed model unites and inspires above modules by the optimization of interpretability loss function *L*_*in*_.

### 3.2. Optimization

The interpretability loss function *L*_*in*_ includes three sub-terms: symmetric loss *L*_*sym*_, apposition loss *L*_*ap*_, and non-negative constraint loss *L*_*nc*_, as shown in Figure 1.

The symmetric loss *L*_*sym*_ is used for guiding the supervisor network to reconstruct original input data. Let the supervisor network consist of the symmetric encoder and decoder, the symmetric loss *L*_*sym*_ is computed as follows:

(4) $$L_{sym}=\sum_{i=1}^{N}{\left\|x_{i}-y_{i}\right\|}_{2}^{2}$$

where *N* is the number of data and ||·||_2_ represents the Euclidean norm. **x**_*i*_ represents the *i*-th original data, and **y**_*i*_ represents the *i*-th reconstructed data. With the minimization of the symmetric loss *L*_*sym*_, the reconstructed data are changed to be the same as the original data.

With the help of the symmetric loss *L*_*sym*_, the supervisor network can learn from reconstructed data, while with the help of the apposition loss *L*_*ap*_, the student network can learn from appositive features. There are *L*+2 layers in the student network. So the apposition loss *L*_*ap*_ has the form as follows:

(5) $$L_{ap}=\sum_{i\;=\;1}^{N}\left(\sum_{l\;=\;1}^{L/2}{\left\|h_{i}^{\left(l\right)}-{\tilde{h}}_{i}^{\left(l\right)}\right\|}_{2}^{2}+\sum_{l\;=\;L/2+1}^{L}{\left\|h_{i}^{\left(l\right)}-{\tilde{h}}_{i}^{\left(l+2\right)}\right\|}_{2}^{2}\right)$$

where ${\tilde{\text{x}}}_{i}^{\left(l\right)}$ represents the output feature of the *l*-th layer of the student network corresponding to *i*-th input data **x**_*i*_. ${\text{x}}_{i}={\tilde{\text{x}}}_{i}^{\left(0\right)}$. By this way, the supervisor network plays a role of supervisor that imparts knowledge of latent information and structure in data to the student network. Thus, the student network learns the patterns of noise, which is not at the expense of inherent knowledge.

The output of encoder is usually not the same as the input of decoder in the student network. The difference of them is included in the non-negative constraint loss *L*_*nc*_ with the formula as follows:

(6) $$\begin{array}{l} L_{nc}=\sum_{i=1}^{N}{\left\|X_{i}-{\hat{X}}_{i}\right\|}_{F}^{2}=\sum_{i=1}^{N}{\left\|X_{i}-ReLU(WH_{i})\right\|}_{F}^{2}\\ \;=\sum_{i=1}^{N}{\left\|X_{i}-ReLU(W\sigma (W^{\prime }X_{i}))\right\|}_{F}^{2} \end{array}$$

Thus, the interpretability loss function *L*_*in*_ is the weighted sum of three subterms in three part of the proposed model:

(7) $$L_{in}=\alpha L_{sym}+\beta L_{ap}+\gamma L_{nc}$$

where α, β, and γ are the hyperparameters adjusting the influence of the symmetric loss *L*_*sym*_, the apposition loss *L*_*ap*_ and the non-negative constraint loss *L*_*nc*_.

By applying the chain rule, the back propagation algorithm computes the update for each model parameter θ. Then the gradient descent algorithm is conducted to decide the new values of each parameter θ as follows:

(8) $$\theta =\theta -\rho \nabla_{\theta }L_{in}$$

where ρ is the learning rate of gradient descent algorithm. The bigger ρ is, the farther parameter goes to the direction of negative gradient of the interpretability loss *L*_*in*_.

For the optimization of parameters in the supervisor network, only the symmetric loss *L*_*sym*_ is considered. That is decided by the supervisor role of supervisor network. Thus, for the parameter θ^(*l*)^ of the *l*-th layer, the corresponding gradient is computed as:

(9) $$\nabla_{\theta^{\left(l\right)}}L_{in}=\alpha \nabla_{\theta^{\left(l\right)}}L_{sym}=\alpha \nabla_{\theta^{\left(l\right)}}h^{\left(l\right)}\nabla_{h^{\left(l\right)}}L_{sym}$$

(10) $$\nabla_{\theta^{\left(l\right)}}h^{\left(l\right)}=\{\begin{array}{c} {\left(h^{\left(l-1\right)}\right)}^{T}◦f^{\prime }(W^{\left(l\right)}h^{\left(l-1\right)}+b^{\left(l\right)}),\theta^{\left(l\right)}=W^{\left(l\right)}\\ f^{\prime }(W^{\left(l\right)}h^{\left(l-1\right)}+b^{\left(l\right)}),\theta^{\left(l\right)}=b^{\left(l\right)} \end{array}$$

(11) $$\nabla_{h^{\left(l\right)}}L_{sym}=\{\begin{array}{c} \nabla_{h^{\left(l\right)}}h^{\left(l+1\right)}\nabla_{h^{\left(l+1\right)}}L_{sym},l<L\\ 2\sum_{i=1}^{N}\left(y_{i}-x_{i}\right),l=L \end{array}$$

(12) $$\nabla_{h^{\left(l\right)}}h^{\left(l+1\right)}={\left(W^{\left(l+1\right)}\right)}^{T}f^{\prime }(W^{\left(l+1\right)}h^{\left(l\right)}+b^{\left(l+1\right)})$$

where **h**^(*l*)^ is the output feature of the *l*-th layer. *f*′[**W**^(*l*)^**h**^(*l*−1)^ + **b**^(*l*)^] represents a diagonal matrix whose diagonal elements are the derivative values of the activation function in the *l*-th layer. *T* means the matrix transposition and ◦ means the operator of output product. When θ^(*l*)^ represents the bias vector **b**^(*l*)^, the gradient $\nabla_{\theta^{\left(l\right)}}h^{\left(l\right)}$ is a matrix whose size is corresponding to the size of **h**^(*l*)^ and **b**^(*l*)^, which is equal to *f*′[**W**^(*l*)^**h**^(*l*−1)^ + **b**^(*l*)^]. When θ^(*l*)^ represents the weight matrix **W**^(*l*)^, $\nabla_{\theta^{\left(l\right)}}h^{\left(l\right)}$ is a three-order tensor whose size is corresponding to the size of **h**^(*l*)^ and **W**^(*l*)^, which is represented by the output product of **h**^(*l*−1)^ and *f*′[**W**^(*l*)^**h**^(*l*−1)^ + **b**^(*l*)^].

For the optimization of parameters in the student network, the apposition loss *L*_*ap*_ and the non-negative constraint loss *L*_*nc*_ are used. The gradient corresponding to the parameter θ^(*l*)^ of the *l*-th layer is computed as:

(13) $$\nabla_{\theta^{\left(l\right)}}L_{in}=\nabla_{\theta^{\left(l\right)}}{\tilde{h}}^{\left(l\right)}\nabla_{{\tilde{h}}^{\left(l\right)}}(\beta L_{ap}+\gamma L_{nc})$$

(14) $$\nabla_{\theta^{\left(l\right)}}{\tilde{h}}^{\left(l\right)}=\{\begin{array}{c} {\left({\tilde{h}}^{\left(l-1\right)}\right)}^{T}◦f^{\prime }(W^{\left(l\right)}{\tilde{x}}^{\left(l-1\right)}+b^{\left(l\right)}),\theta^{\left(l\right)}=W^{\left(l\right)}\\ f^{\prime }(W^{\left(l\right)}{\tilde{h}}^{\left(l-1\right)}+b^{\left(l\right)}),\theta^{\left(l\right)}=b^{\left(l\right)} \end{array}$$

(15) $$\begin{array}{l} \nabla_{{\tilde{h}}^{\left(l\right)}}(\beta L_{ap}+\gamma L_{nc})=Grad({\tilde{h}}^{\left(l\right)})\\ +\{\begin{array}{c} \nabla_{{\tilde{h}}^{\left(l\right)}}{\tilde{h}}^{\left(l+1\right)}\nabla_{{\tilde{h}}^{\left(l+1\right)}}(\beta L_{ap}+\gamma L_{nc}),l<L+2\\ 2\beta \sum_{i\;=\;1}^{N}\left({\tilde{y}}_{i}-y_{i}\right),l=L+2 \end{array} \end{array}$$

(16) $$\nabla_{{\tilde{h}}^{\left(l\right)}}{\tilde{h}}^{\left(l+1\right)}={\left(W^{\left(l+1\right)}\right)}^{T}f^{\prime }(W^{\left(l+1\right)}{\tilde{h}}^{\left(l\right)}+b^{\left(l+1\right)})$$

where ${\tilde{\text{h}}}^{\left(l\right)}$ is the output feature of the *l*-th renumbered layer in the student network. $\nabla_{{\tilde{\text{h}}}^{\left(l\right)}}\left(\beta L_{ap}+\gamma L_{nc}\right)$ includes $Grad\left[{\tilde{\text{h}}}^{\left(l\right)}\right]$, the gradient from loss function directly which is depended on the layer itself, and the gradient transmitted from the next layer. Mentioned that the NMF module is without the bias.

Finally, the overall training algorithm are outlined in the Algorithm 1.

**Algorithm 1:** Optimization algorithm of DDNMF

**Input:** input dataset **X**, noisy dataset $\tilde{\text{X}}$, hyper parameters
{α, β, γ}
Initialize randomly trainable parameters
**for** not converged **do**
**for** each epoch of data {*x*} and $\left\{\tilde{\text{x}}\right\}$ **do**
forward feed in the supervisor network with Equation (6)
forward feed in the student network with Equations (6, 8)
compute the losses with Equations (9)–(12)
update parameters in the supervisor network with Equations (14)–(17)
update parameters in the student network with Equations (18)–(21)
**end for**
**end for**
**Output:** trained network

### 3.3. Algorithm Complexity

Both of input dataset **X** and noisy dataset $\tilde{\text{X}}$ of DDNMF have *N* samples. Assume that the overall training algorithm is converged after going through the whole datasets *t* times, the number of iterations is *O*(*tN*). In each iteration, samples from the two datasets are input into the supervisor network and the student network. The supervisor network has *L* layers, and the student network contains an extra NMF module with a fixed number of layers. Thus, the total layer depth of DDNMF is *O*(*L*). In each layer, the forward feed and back propagation process are conducted to extract features and modify parameters. Assume that the maximum dimension of features in all layers is *D*, the space complexity, also the number of parameters including *W* and *b*, will be *O*(*D*^2^) in each layer. In forward feed process, both of the multiplication and addition operation on parameters in each layer are *O*(*D*^2^), and the output of activation function is computed *O*(*D*) times. Similarly, in back propagation process, the number of multiplication/addition operation and derivative function computation in a layer are *O*(*D*^2^) and *O*(*D*), respectively. So the time complexity of each layer are also *O*(*D*^2^). Considering that in each iteration, the time complexity of the calculation on loss terms *L*_*sym*_, *L*_*nc*_, and *L*_*ap*_ are *O*(*D*), *O*(*D*), *O*(*LD*), respectively, the time complexity of loss can be ignored in the analysis of algorithm complexity. In conclusion, the space complexity of DDNMF is *O*(*LD*^2^), and the time complexity of DDNMF is *O*(*tNLD*^2^).

## 4. Experiments

In this section, the performance of DDNMF is extensively evaluated. In the clustering task on several datasets, DDNMF is compared with various methods in terms of accuracy (ACC), Normalized Mutual Information (NMI), and Adjusted Rand Index (ARI). Results show that DDNMF outperforms the comparative methods. Implemented by Python, all the codes are performed on the Dell PowerEdge R740 server with a Tesla V100 GPU, Intel Xeon Silver 4114 CPU (2.20 GHz) and 256 GB DDR4 memory. The details of experiments are described in the following subsections.

### 4.1. Datasets

MNIST and Fashion-MNIST datasets are used to verify the performance of DDNMF.

MINST (Salakhutdinov and Murray, 2008) consists of 70,000 labeled gray images with the size of 28×28, which describe the handwritten digits from 0 to 9. The experiments use the images to conduct DDNMF and comparative methods, measuring the performance by labels.

FashionMNIST (Xiao et al., 2017) consists of 70,000 gray 28×28 images with 10 different labels, such as trouser, coat, and sneaker. Similarly, images are used for comparative methods and labels are applied for evaluation.

### 4.2. Comparative Methods

**K-means** is a basic clustering method widely used in various tasks. In our experiment, it plays the role of not only a baseline but also a component of other comparative methods.

**Gaussian Mixture Model (GMM)**, as a generative method, is also a typical clustering method based on the probability theory. Like K-means, GMM can also cluster the extracted representations to verify the performance of comparative methods in the experiment.

**Autoencoder** is a kind of normal unsupervised deep non-linear feature extractor. There are two autoencoders with different parameters settings in our experiment, denoted as AE1 and AE2. Among them, AE1 has the same number of layers and neurons with the proposed method, and the depth of AE2 is one layer less than AE1. The ReLU activation function is employed in both of the two autoencoders.

**NMF** is a well-known matrix factorization methods, implemented as a comparative component to extract good representations in the experiment.

### 4.3. Evaluation Metrics

ACC, NMI, and ARI are employed as evaluation metrics in the experiment.

ACC usually measures the average correct rate of classification. By the Kuhn-Munkres algorithm, ACC can also be used in the evaluation of clustering.

(17) $$ACC=\frac{1}{N}\sum_{i=1}^{N}\delta (a_{i},km(b_{i}))$$

where *N* is the number of the data. δ(*x, y*) equals to 1 if and only if *x* = *y*. *a*_*i*_ and *b*_*i*_ are the real and the predicted *i*-th clustering assignment. *km*() is the mapping decided by the Kuhn-Munkres algorithm.

NMI is defined as:

(18) $$NMI=\frac{2I(A,B)}{H(A)+H(B)}$$

where *I*(*A, B*) is the mutual information between clustering assignment *A* and *B*. *H*(*A*) is the entropy of *A*.

ARI is based on Rand Index (RI):

(19) $$ARI=\frac{RI-E(RI)}{\max(RI)-E(RI)},RI=\frac{a+d}{C_{N}^{2}}$$

where *E*() represents the expectation. $C_{N}^{2}$ is the combinatorial number of pairing the samples. *RI* is the rate of correct pairs including *a* same label with same clustering and *d* different labels with different clusterings.

### 4.4. Experimental Results

Tables 1, 2 are the experimental results in terms of ACC, NMI, and ARI of comparative methods on MNIST and FashionMNIST datasets. There are some main observations and discussions as follows:

* Experiments on the features extracted by AE2 (lines 5–8 of the two tables) outperforms experiments on original data (line 1–4 of the two tables). The increase of ACC, NMI, and ARI shows that AE2 can learn deep representations which concentrate informative knowledge of data and are more suitable for clustering task than original data.
* It is shown that conducting NMF on both of original data (line 3–4 of the two tables) and deep representations (lines 7–8 of the two tables) for interpretability will decrease the performance of clustering at most case. That is, combining NMF and data directly will degrade the performance of representations because of linear decomposition and the decrease of matrix rank.
* Compared with K-means (odd lines of the two tables), GMM (even lines of the two tables) usually clusters data or features better. This is because K-means could be regarded as a variant of GMM with the sphere constraint that its covariance matrix must be like the identity matrix. Which simplifies the procedure of K-means but limits its ability of fitting data distribution.
* By adding NMF module into the process of extracting features and training in the end-to-end manner, our DDNMF achieves the best performance in terms of ACC, NMI, and ARI on both of MNIST and FashionMNIST datasets. Which demonstrates the superiority of the proposed method.

**Table 1:** The experimental results on MNIST.

**Comparative methods**	**ACC**	**NMI**	**ARI**
K-means	0.55480	0.51894	0.38776
GMM	0.56040	0.53567	0.40968
NMF+K-means	0.51560	0.42729	0.30529
NMF+GMM	0.55180	0.43959	0.32773
AE2+K-means	0.58850	0.52520	0.40667
AE2+GMM	0.63030	0.55528	0.44391
AE2+NMF+K-means	0.53250	0.49350	0.37485
AE2+NMF+GMM	0.57320	0.49281	0.38625
DDNMF+K-means	**0.81150**	**0.70153**	**0.64887**
DDNMF+GMM	**0.74840**	**0.65667**	**0.57424**

*The best two values are in bold*.

**Table 2:** The experimental results on FashionMNIST.

**Comparative methods**	**ACC**	**NMI**	**ARI**
K-means	0.48730	0.51603	0.35172
GMM	0.52260	0.51377	0.35381
NMF+K-means	0.52990	0.53305	0.36654
NMF+GMM	0.54220	0.50626	0.33398
AE2+K-means	0.53860	0.53587	0.38220
AE2+GMM	0.58540	0.49864	0.36436
AE2+NMF+K-means	0.55380	0.55150	0.38742
AE2+NMF+GMM	0.56370	0.52247	0.36366
DDNMF+K-means	**0.63120**	**0.59671**	**0.46759**
DDNMF+GMM	**0.65470**	**0.60651**	**0.47403**

*The best two values are in bold*.

**Figure 2:**
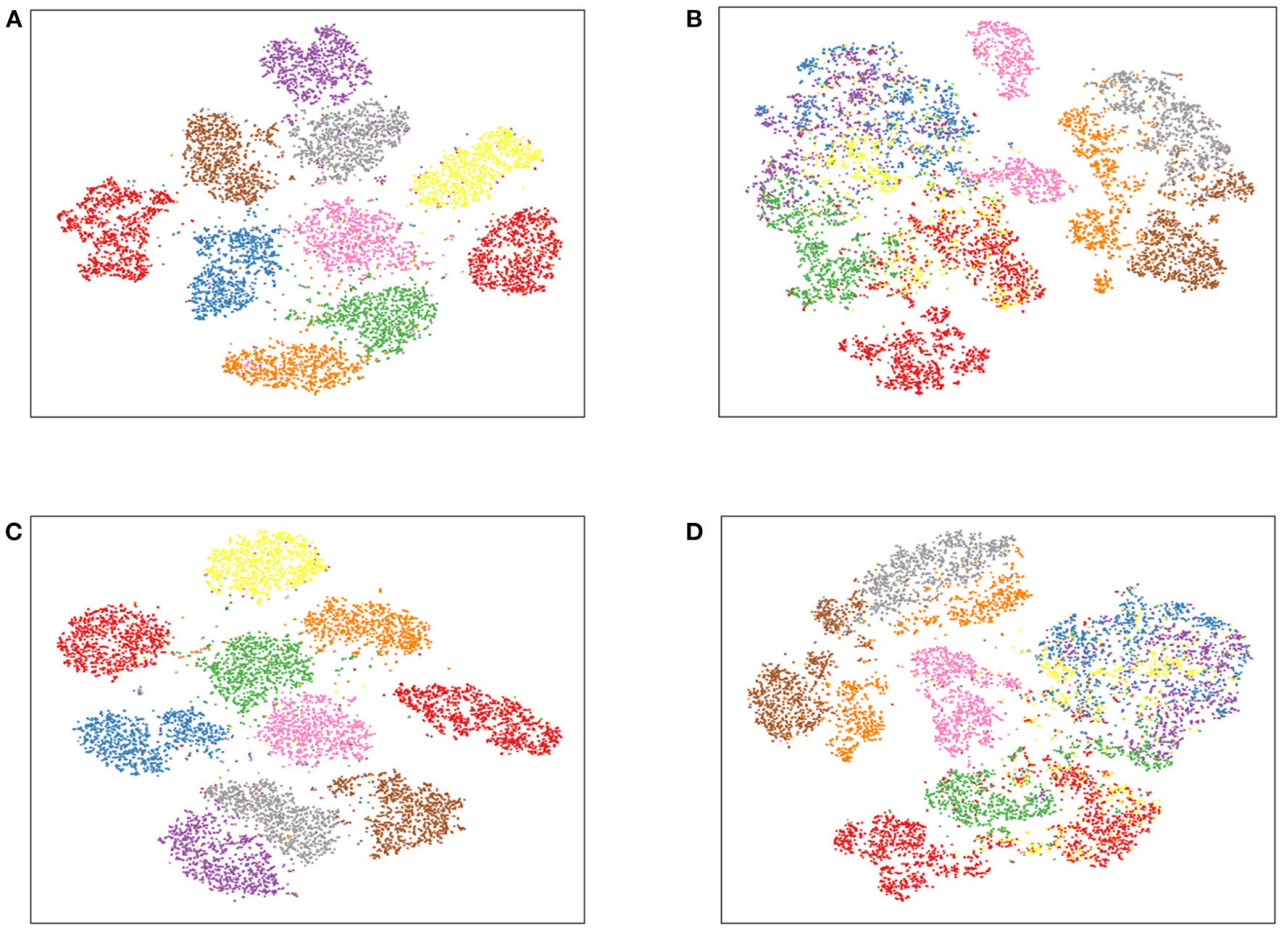
The t-SNE figures of DDNMF and AE1 on MNIST and FashionMNIST. **(A)** DDNMF+MNIST, **(B)** DDNMF+FashionMNIST, **(C)** AE1+MNIST, **(D)** AE1+FashionMNIST.

Figure 2 shows the t-distributed Stochastic Neighbor Embedding (t-SNE) figures of DDNMF and AE1 on two datasets. The visualization also exhibits the good performance of DDNMF.

**Figure 3:**
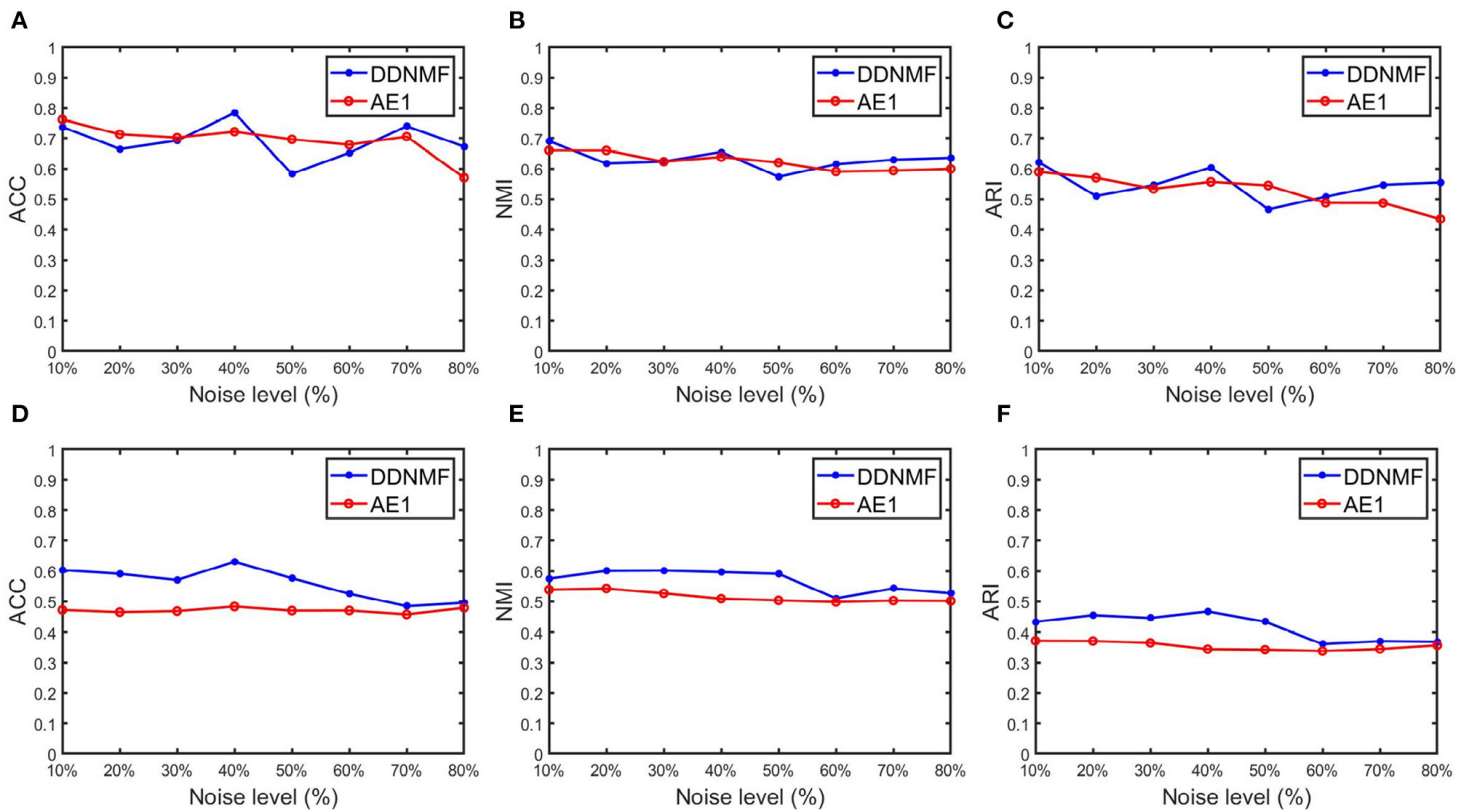
The changes of clustering performance of DDNMF and AE1 on different noise levels. **(A–C)** Results on the MNIST dataset, and **(D–F)** are on the FashionMNIST dataset.

To verify the performance of DDNMF in data denoising, experiments on two datasets with noise from 10 to 80% are conducted. Table 3 and Figure 3 are the changes of clustering performance of DDNMF and AE1 on different noise levels. It shows that both of DDNMF and AE1 suffer from the increase of the noise level, and with the increase of noise, DDNMF reveals its advantage of denoising and achieves the better performance than AE1 on the two datasets. On the MNIST dataset, DDNMF has the higher ACC, NMI, and ARI than AE1 when the noise level is high enough. On the FashionMNIST dataset, DDNMF always outperforms AE1 on all metrics. Which demonstrates that with low noise levels, DDNMF can achieve comparable or even better performance than AE1, the autoencoder with the same number of layers and neurons, with the help of the supervisor network. And with high noise levels, DDNMF can address the challenge of noise by the denoising property of the student network. Thus, experiments on the two datasets with different noise levels verify the effectiveness of the proposed DDNMF.

**Table 3:** The results of DDNMF and AE1 on different noise levels.

**Noise level**	**10%**	**20%**	**30%**	**40%**	**50%**	**60%**	**70%**	**80%**
MNIST+DDNMF:ACC	0.73810	0.66530	0.69270	0.78400	0.58310	0.65280	0.74060	0.67260
MNIST+AE1:ACC	0.76220	0.71380	0.70260	0.72230	0.69670	0.67900	0.70590	0.57040
MNIST+DDNMF:NMI	0.69090	0.61762	0.62424	0.65516	0.57338	0.61489	0.63023	0.63649
MNIST+AE1:NMI	0.66079	0.66039	0.62352	0.63923	0.62091	0.59071	0.59371	0.59938
MNIST+DDNMF:ARI	0.62203	0.51083	0.54637	0.60423	0.46580	0.50822	0.54740	0.55506
MNIST+AE1:ARI	0.58954	0.57022	0.53432	0.55698	0.54528	0.48758	0.48707	0.43513
FashionMNIST+DDNMF:ACC	0.60360	0.59130	0.57120	0.63120	0.57690	0.52600	0.48480	0.49590
FashionMNIST+AE1:ACC	0.47270	0.46550	0.46890	0.48380	0.47050	0.47090	0.45800	0.47970
FashionMNIST+DDNMF:NMI	0.57559	0.60082	0.60209	0.59671	0.59133	0.51081	0.54363	0.52765
FashionMNIST+AE1:NMI	0.53955	0.54333	0.52740	0.50946	0.50394	0.49874	0.50315	0.50206
FashionMNIST+DDNMF:ARI	0.43383	0.45541	0.44640	0.46759	0.43419	0.36124	0.37084	0.36899
FashionMNIST+AE1:ARI	0.37198	0.37127	0.36532	0.34385	0.34220	0.33748	0.34438	0.35684

## 5. Conclusion

In this paper, we proposed DDNMF for learning deep denoising interpretable representations. The end-to-end deep architecture composed of a supervisor network learning noise-free knowledge and a student network capturing part-based representation of non-negative constraints is introduced, mining data with noise. The interpretability loss is designed to distill interpretable knowledge of real-world noisy data. Experimental results on standard datasets demonstrated the effectiveness of DDNMF. In the future, on the basis of the proposed basic deep architecture, more sophisticated deep matrix factorization method will be exploited for deep representations of multimodal data.

## Data Availability Statement

Publicly available datasets were analyzed in this study. This data can be found at: https://tensorflow.google.cn/datasets/catalog/binarized_mnist; https://tensorflow.google.cn/datasets/catalog/fashion_mnist.

## Author Contributions

ZC designed the overall architecture and wrote the part of Introduction and Method. SJ wrote the rest of this article. RL and JZ conducted the experiments to evaluate the performance of the proposed method. All authors contributed to the article and approved the submitted version.

## Conflict of Interest

The authors declare that the research was conducted in the absence of any commercial or financial relationships that could be construed as a potential conflict of interest.
